# The TIP30 Protein Complex, Arachidonic Acid and Coenzyme A Are Required for Vesicle Membrane Fusion

**DOI:** 10.1371/journal.pone.0021233

**Published:** 2011-06-24

**Authors:** Chengliang Zhang, Aimin Li, Shenglan Gao, Xinchun Zhang, Hua Xiao

**Affiliations:** 1 Department of Biomedical and Integrative Physiology, Michigan State University, East Lansing, Michigan, United States of America; 2 Genetics Program, Michigan State University, East Lansing, Michigan, United States of America; 3 Department of Oncology, Nanfang Hospital, Southern Medical University, Guangzhou, Guangdong, People's Republic of China; Institut Européen de Chimie et Biologie, France

## Abstract

Efficient membrane fusion has been successfully mimicked *in vitro* using artificial membranes and a number of cellular proteins that are currently known to participate in membrane fusion. However, these proteins are not sufficient to promote efficient fusion between biological membranes, indicating that critical fusogenic factors remain unidentified. We have recently identified a TIP30 protein complex containing TIP30, acyl-CoA synthetase long-chain family member 4 (ACSL4) and Endophilin B1 (Endo B1) that promotes the fusion of endocytic vesicles with Rab5a vesicles, which transport endosomal acidification enzymes vacuolar (H^+^)-ATPases (V-ATPases) to the early endosomes *in vivo*. Here, we demonstrate that the TIP30 protein complex facilitates the fusion of endocytic vesicles with Rab5a vesicles *in vitro*. Fusion of the two vesicles also depends on arachidonic acid, coenzyme A and the synthesis of arachidonyl-CoA by ACSL4. Moreover, the TIP30 complex is able to transfer arachidonyl groups onto phosphatidic acid (PA), producing a new lipid species that is capable of inducing close contact between membranes. Together, our data suggest that the TIP30 complex facilitates biological membrane fusion through modification of PA on membranes.

## Introduction

Membrane fusion is well known as one of the most fundamental cellular processes in living organisms. It generally requires cellular factors to bring donor and recipient membranes into close proximity, increase membrane curvature, and disturb lipid bilayers [1], [2]. Extensive studies during the past decades have led to the identification of a number of fusogenic proteins and lipids. In addition to lipids such as acyl-CoA [3], [4], arachidonic acid [5], [6], phosphoinositides, phosphotidic acid and diacylglycerol [7], [8], [9], many proteins including Rab5, Rab5 effectors, SNARE proteins and SNARE accessory factors are necessary for various membrane fusion events [10], [11].

Recently, efficient endosome fusion was successfully mimicked using proteoliposomes reconstituted with 17 recombinant core fusion proteins [2], [12]. Notably, however, these proteins are not sufficient to promote efficient fusion between biological membranes, indicating that biological membrane fusion requires more cellular factors than artificial membrane fusion. Given that purified endosomes contain the core SNARE proteins Syntaxin6, Syntaxin13, Vti1a and VAMP4, it is likely that factors needed for initiating membrane fusion have yet been identified.

Among the lipids that play key roles in membrane fusion, arachidonic acid has been shown to be required for endosome fusion *in vitro* [6], and is the most effective fusogen in chromaffin granule fusion [13]. In addition, membrane-bound arachidonic acid can drive annexin II-mediated membrane fusion of the lamellar body with the plasma membrane during the exocytosis [5].

We have recently identified a protein complex containing TIP30, ACSL4 and Endo B1 that interacts with Rab5a and facilitates the loading of V-ATPases on endocytics vesicles. Inhibiting any of these proteins causes the mislocalization of V-ATPases, thus leading to the trapping of EGF-EGFR complexes in endocytic vesicles, delayed EGFR degradation and sustained EGFR endosomal signaling [14]. In addition, we have shown that Rab5a and V-ATPase reside in vesicles devoid of EGFR, the early endosomal marker EEA1 and the recycling endosomal marker transferrin receptor (TfR), suggesting that Rab5a functions as a identity tag for vesicles that deliver V-ATPases to endosomes [14]. Given that localization of integral membrane proteins to their target membranes requires vesicle membrane fusion [15], we therefore examined if the TIP30 protein complex can promote vesicle membrane fusion *in vitro*. Since ACSL4 was identified in the protein complex and it is an acyl-CoA synthetase that prefers arachidonic acid as the reaction substrate, we included both arachidonic acid and coenzyme A in the *in vitro* membrane fusion reactions. We found that the TIP30 complex and the synthesis of arachidonyl-CoA from arachidonic acid and coenzyme A are required for efficient fusion of Rab5a vesicles with endocytic vesicles. The TIP30 complex may initiate membrane fusion by modifying membrane PA.

## Results

### The TIP30 complex, arachidonic acid and coenzyme A promote fusion between endocytic and Rab5a vesicles

To investigate whether the TIP30 complex can promote membrane fusion *in vitro*, we used a confocal microscopy-based *in vitro* fusion assay [16], [17] to monitor fusion between endocytic and Rab5a vesicles in an initial pilot experiment. Endocytic and Rab5a vesicles were prepared from HepG2 cells that do not contain detectable endogenous TIP30 and EGFR. Rab5a vesicles were labeled by expressing EYFP-Rab5a fusion proteins and prepared from serum-starved cells. Endocytic vesicles were labeled by expressing EGFR-DsRed fusion proteins and prepared from EGF treated cells. The two types of vesicles that contain equal amount of proteins were incubated in the fusion buffer at 37°C followed by examination with confocal microscopy. Vesicle fusion and aggregation were represented by the fluorescence overlap between EGFR-DsRed and EYFP-Rab5a.

**Figure 1:**
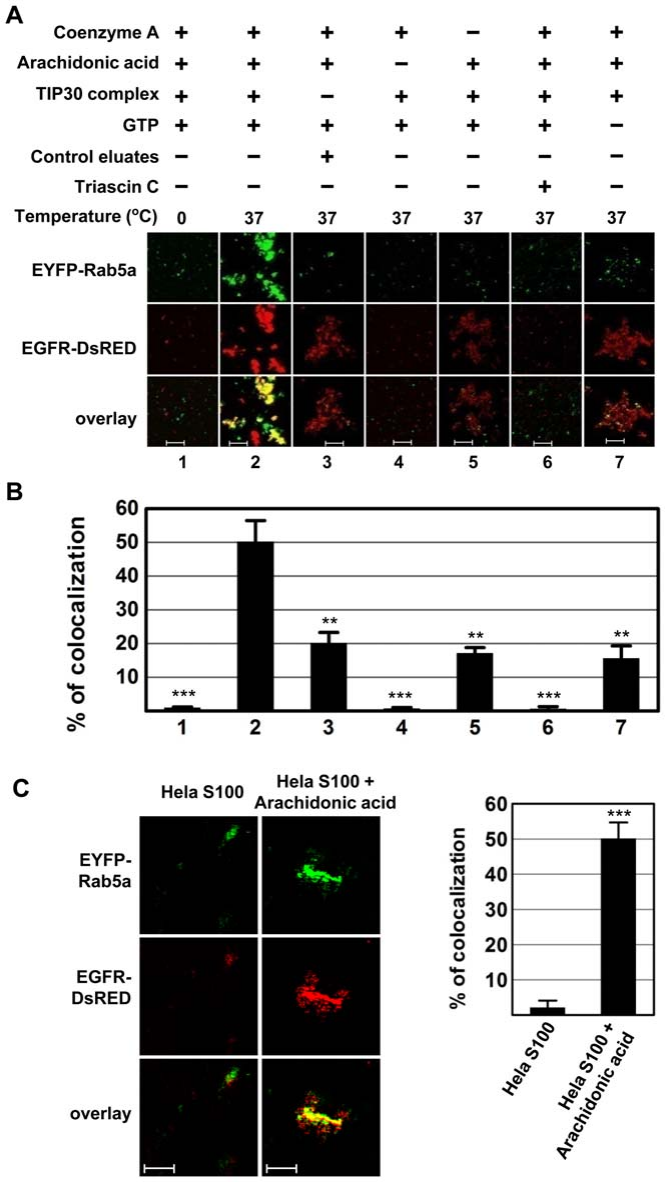
The TIP30 protein complex, arachidonic acid and coenzyme A promote the fusion between endocytic and Rab5a vesicles. (**A**) Aliquots of isolated EGFR-DsRed and EYFP-Rab5a vesicles (both contain 20 µg of proteins) were mixed and incubated in reactions (20 µl) with the indicated components. The resulting fusion products were spotted on glass slides and images were taken using confocal microscope. Panels 1, 4 and 6 were scanned with 3× amplification gain setting due to lower fluorescence intensity of individual vesicles. Arachidonic acid (100 nmol) was used in the reactions. Images are single plane and are representative for at least three independent experiments. Scale bars, 5 µm. (**B**) Signal overlap was quantified using MBF_ImageJ. Pearson's colocalization coefficients were calculated from three independent experiments and were converted to percentages. Data represent means ± SEM. **P<0.01, ***P<0.001; t test. (**C**) Arachidonic acid promotes the vesicle fusion induced by HeLa cell S100. S100 fractions (4 mg/ml) of HeLa cells were incubated with isolated EGFR-DsRed and EYFP-Rab5a vesicles (both contain 20 µg proteins) in the absence or presence of 100 nmol arachidonic acid. Resulting vesicles were examined using confocal microscopy (left panel) and fluorescence overlaps were quantified (right panel).

Since ACSL4 is an acyl-CoA ligase with high substrate preference for arachidonic acid (C20:4) [18], we first tested if arachidonic acid and coenzyme A are needed. Vesicles resulting from reactions that were kept on ice were evenly distributed on the slides, appearing as small vesicles with low fluorescence intensity and no fluorescence overlap (Figure 1A, lane 1; Figure 1B). Similarly, no fluorescence overlap was observed in the absence of arachidonic acid or in the presence of triacsin C (10 uM), a potent inhibitor of ACSL4 (Figure 1A, lanes 4 and 6; Figure 1B). In contrast, in the presence of arachidonic acid and coenzyme A, immunopurified TIP30 complex caused vesicle enlargement and significantly increased fluorescence overlap (TIP30 complex: 50±6%; control eluates: 20±3%; omitting coenzyme A: 17±2%; omitting GTP: 16±4%; n = 6 representative confocal images of 143×143 µm, p<0.01 versus TIP30 complex; Figure 1A and 1B), thereby resulting in much intensified fluorescence. Rab5a vesicles attached to aggregated endocytic vesicles, but remained as small particles when the TIP30 complex, coenzyme A, or GTP was omitted. The effect of GTP exclusion is consistent with the fact that GTP is a known membrane fusion factor and required for Rab5a function. We next screened for other fatty acids that might promote membrane fusion, including palmitic, palmitoleic, oleic, linoleic, linolenic, eicosapentaenoic, and docosahexaenoic acids. None of these fatty acids could promote vesicle enlargement and fluorescence overlap (data not shown). Furthermore, arachidonic acid significantly increased fluorescence overlap in the presence of HeLa cell cytoplasmic S100 extracts that containing the TIP30 complex (HeLa S100: 2±2%; HeLa S100+arachidonic acid: 50±5%; n = 6 representative confocal images of 143×143 µm, p<0.01 versus HeLa S100; Figure 1C). These results suggest that arachidonic acid and the TIP30 complex are involved in membrane aggregation and/or fusion, which are consistent with our *in vivo* data [14] and previous reports that arachidonic acid is essential for vesicle membrane fusion [6], [13].

**Figure 2:**
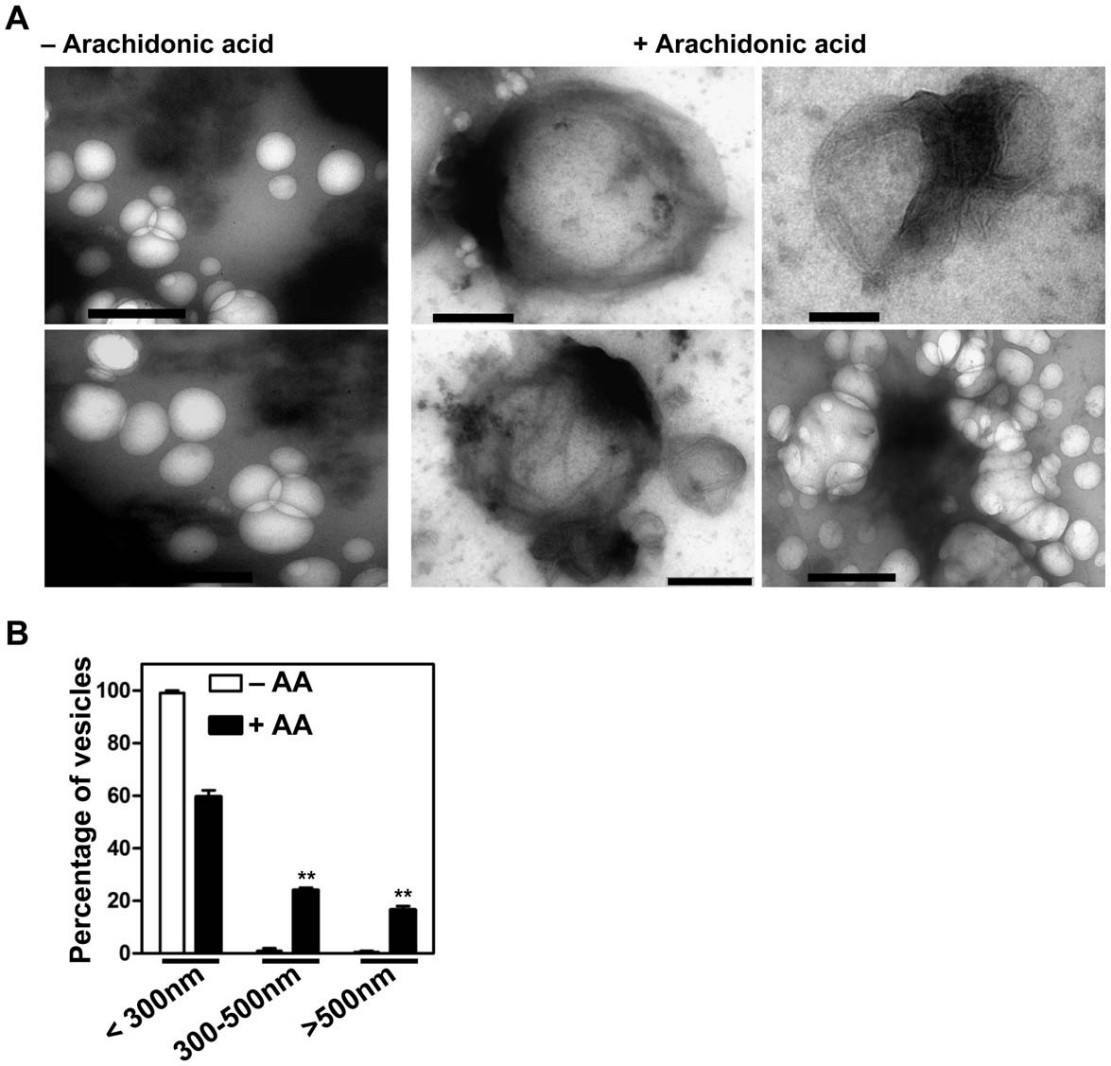
Transmission electron microscopy analysis of products from in vitro vesicle fusion assays. (**A**) EGFR-DsRed and EYFP-Rab5a vesicles were incubated with immunopurified TIP30 complex in the fusion buffer with (right panel) or without (left panel) 100 nmol of arachidonic acid. Resulting vesicles were stained with uranyl acetate and examined using TEM. Scale bars, 500 nm. (**B**) The graphs show the percentages of vesicles with different diameters. At least 6 images from two independent experiments were counted. Data represent means ± SEM. n = 150; **P<0.01, t test.

To determine whether that vesicle enlargement and fluorescence overlap were due to membrane fusion or aggregation, we examined the vesicles that resulted from fusion reactions by transmission electron microscopy (TEM). Without the addition of arachidonic acid, all vesicles appeared spherical and ranged in diameter from 50 to 300 nm. In contrast, the complete fusion reaction led to the production of vesicles enlarged to more than 1 µm in diameter (Figure 2A); the larger vesicles were not found in reactions without arachidonic acid. Ongoing fusion could also be observed. Quantitative analysis revealed a significant increase in the amount of enlarged vesicles (with arachidonic acid: 300–500 nm, 24±2%; >500 nm, 16.5±1.5%; without arachidonic acid: 300–500 nm, 1±0.2%; >500 nm, 0; n = 150, p<0.01; Figure 2B). Given the fluorescent overlap between two types of vesicles in the complete reactions shown in Figure 1, these results indicate that the TIP30 complex and synthesis of arachidonyl-CoA are essential for the fusion of Rab5a vesicles with endocytic vesicles.

### Recombinant TIP30, ACSL4 and Endo B1 can replace the TIP30 complex in promoting fusion of endocytic vesicles with Rab5a vesicles

**Figure 3:**
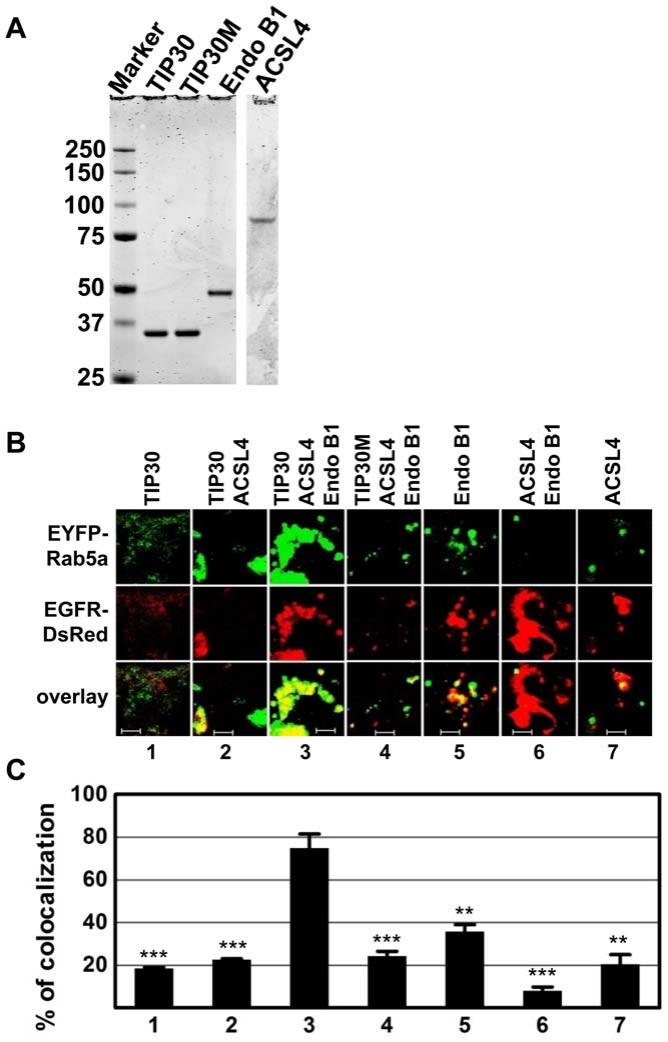
Bacterially expressed recombinant TIP30, ACSL4 and Endo B1 can replace the TIP30 complex in promoting efficient vesicle fusion. (**A**) Recombinant proteins were expressed in BL21 cells and were purified using cobalt affinity resins. Eluted proteins were subjected to SDS-PAGE analysis followed by Coomassie blue staining. Images were acquired using a Li-Cor scanner. (**B**) EGFR-DsRed and EYFP-Rab5a vesicles were incubated with the indicated recombinant proteins (20 ng each) in the fusion buffer. Resulting fusion products were examined using confocal microscope. Scale bars, 5 µm. (**C**) Quantification of images represented in (B). Signal overlap was quantified using MBF_ImageJ. Pearson's colocalization coefficients were calculated from three independent experiments and converted to percentages. Data represent means ± SEM. **P<0.01, ***P<0.001; t test.

To exclude possible influences of other associated or contaminating proteins in immunopurified TIP30 complexes, we replaced the immuno-complexes with bacterially-expressed recombinant TIP30, ACSL4 and Endo B1 in cell free assays. The three highly purified recombinant proteins (Figure 3A) can efficiently promote fluorescence overlap (75±6%), whereas lack of any of these proteins led to significantly less overlap (TIP30: 18±1%; TIP30 and ACSL4: 23±1%; Endo B1: 35±3%; ACSL4 and EndoB1: 8±2%; ACSL4: 20±5%; n = 6 representative images of 143×143 µm; p<0.01 versus TIP30, ACSL4 and Endo B1; Figure 3B and 3C). TIP30M, a TIP30 mutant with a mutated putative nucleotide binding motif (GXXGXXG) [19], only promoted 24±2% overlap (n = 6 representative images of 143×143 µm; p<0.01 versus TIP30, ACSL4 and Endo B1; Figure 3B and 3C, panel 4). These data suggest that TIP30, ACSL4 and Endo B1 together can substitute for the TIP30 complex in promoting the fusion between endocytic and Rab5a vesicles.

### Vesicle tethering and stacking induced by acylation of phosphatidic acid

**Figure 4:**
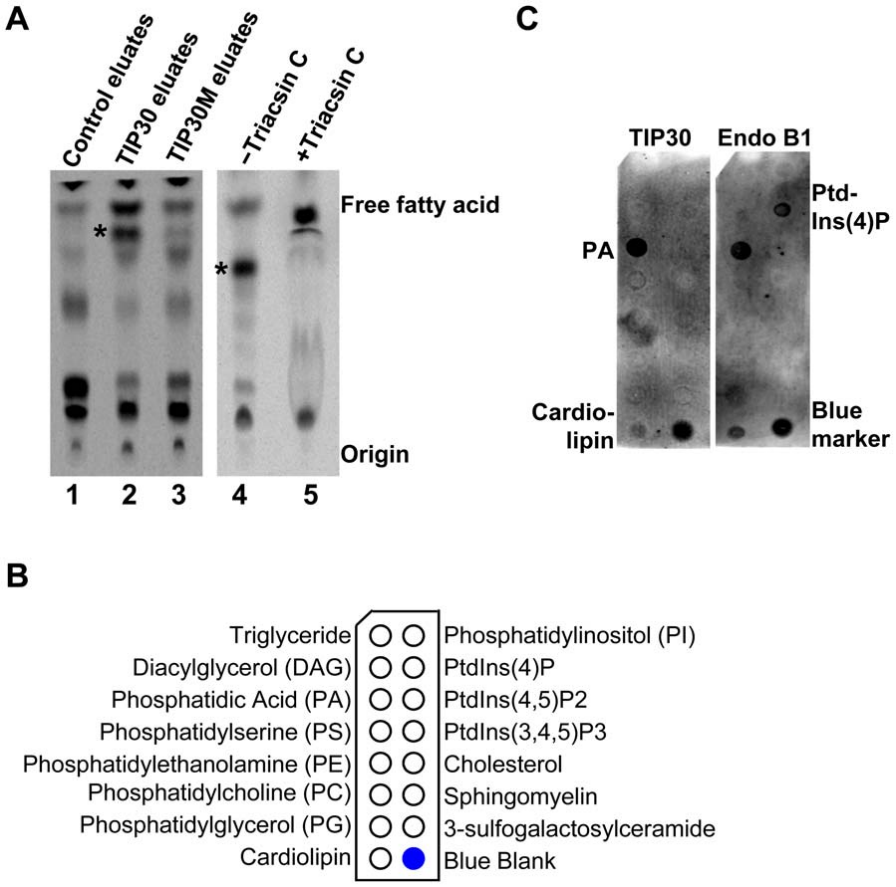
TIP30 and Endo B1 strongly bind phosphatidic acid. (**A**) Fatty acylation of endosomal lipids by the TIP30 complex. [^3^H]-arachidonic acid can be transferred to endosomal membrane lipids by the TIP30 complex, but not by TIP30M immunoprecipitates or control immunoprecipitates (left panel). The transfer was blocked by 10 µM triacsin C (right panel). Image was acquired by scanning lipids resolved on TLC plate with a Molecular Dynamics Storm 860. * indicates the radiolabeled lipid. (**B**) The schematic diagram shows the lipid species pre-spotted on membranes that are used in lipid-protein overlay assays. (**C**) TIP30 and Endo B1 strongly bind phosphatidic acid (PA). Protein-lipid overlay assays were carried out by incubating recombinant proteins with membrane strips containing 15 pre-spotted lipids. Membranes were scanned using a Li-Cor scanner after being sequentially overlaid with primary and fluorescent secondary antibodies.

To gain further insight into how TIP30 and its interacting proteins mediate membrane fusion, we first used ^3^H-arachidonic acid to label membrane lipids and found that at least one lipid species in purified early endosomes was specifically labeled by the TIP30 complex (Figure 4A, lane 2). The production of the labeled lipid was significantly less in the reaction using control eluates (lane 1) or TIP30M immunoprecipitates (lane 3), and was blocked by triacsin C (lane 5). The new lipid species was not observed when Rab5a vesicles were used in the reactions (data not shown). These data indicate that lipids on endocytic vesicles are the specific recipients of the arachidonyl group.

**Figure 5:**
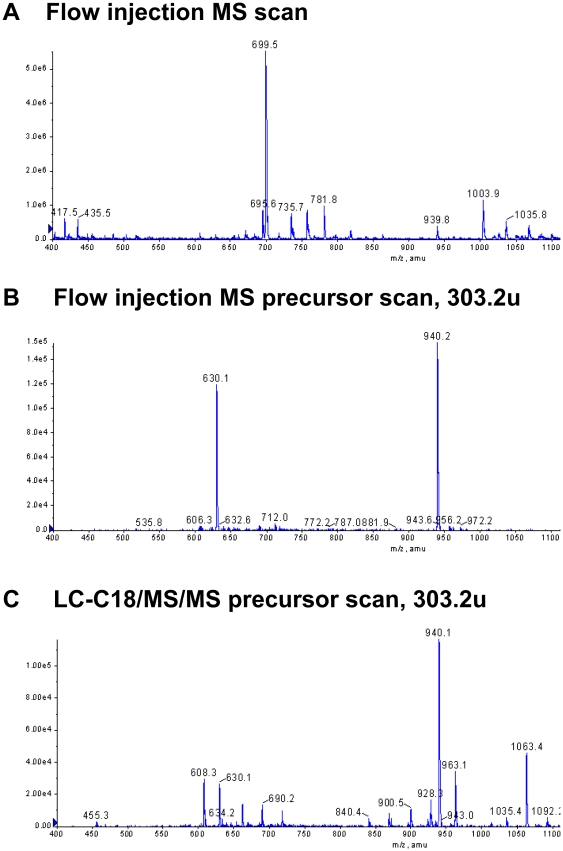
Arachidonyl group was transferred onto phosphatidic acid. (**A**) PA was incubated with the TIP30 protein complex in the fusion buffer. Resulting lipids were purified and subjected to flow injection negative scan in the range of 400–1400 u. A predominant peak of 699.5 was detected. Atomic mass: arachidonic acid, 304.5 u; 18:1 PA, 699.5 u. The lipids in the smaller peaks have molecular weights that do not match any of the expected PA derivatives. (**B**) The flow injection negative precursor scan for 303.2 u over a mass range of 400–1400 u. (**C**) The LC-C18 negative precursor scan for 303.2 u over a mass range of 400–1400 u. The lipids after the acylation reaction was extracted and chromatographed on a C-18 column to reduce possible adduction mechanisms of compounds detected in the flow injection negative precursor scan of 303.2 u.

To identify the lipids that are modified, we performed protein-lipid overlay assays using membrane strips prespotted with 15 cellular membrane lipids (Figure 4B). TIP30 and Endo B1 specifically bind phosphatidic acid (PA) and cardiolipin. Endo B1 also binds phosphatidylinositol 4-phosphate (PtdIns(4)P; Figure 4C). ACSL4 and Rab5a did not bind any lipids spotted on the strips (data not shown). Cardiolipin is found predominantly in the inner mitochondrial membrane, whereas PA has been implicated in the fusion of various intracellular membranes [7], [20]. Therefore, we focused on PA and tested whether the TIP30 complex could convert PA to PA-derivatives. Lipids extracted from the acylation reactions with PA and arachidonic acid as substrates were subjected to MS/MS and LC-MS/MS spectrometry analyses (Figure 5A–5C). The flow injection precursor scan in both modes revealed one predominant peak with an m/z (mass-to-charge ratio) value of 940.2, which does not match the exact mass of any known lipids in the database (lipidmaps.org). Thus, the identity of lipid product of the TIP30 complex remains unknown. However, since its mass is very close to the mass (940.85) of triacylglycerols (C_61_H_112_O_6_), we speculate that it might be a triacylglycerol.

**Figure 6:**
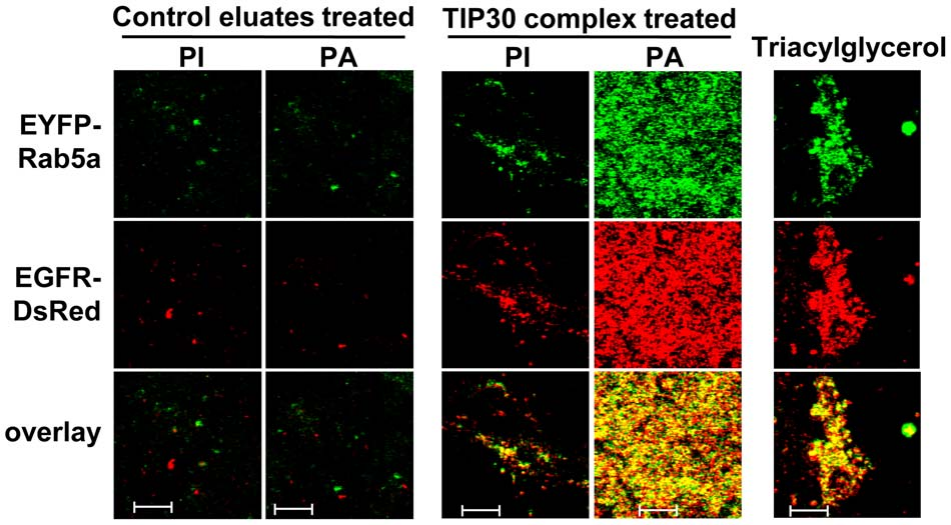
Fatty acylation of phosphatidic acid promotes vesicle aggregation. Lipids were extracted after incubating 100 nmol PA or phosphatidylinositol (PI) with 100 nmol arachidonic acid the TIP30 complex or control eluates. Lipids were resuspended in homogenization buffer by sonication and were mixed with EGFR-DsRed and EYFP-Rab5a vesicles in *in vitro* fusion buffer at 37°C. Resulting vesicles were spotted on glass slides and images were taken using confocal microscope. Scale bars, 5 µm.

To determine how PA derivatives *per se* would affect membrane fusion, we carried out acylation reactions by incubating PA or phosphoinositide (PI) with the TIP30 complex in the fusion buffer. The resulting lipids were purified, resuspended by sonication, and incubated with vesicles. Interestingly, the PA derivatives promoted dramatic fluorescence overlap among endocytic and Rab5a vesicles on ice (Figure 6). In contrast, PA incubated with control eluates did not promote significant fluorescence overlap among the vesicles, nor did phosphoinositide (PI) that was prepared with the same procedure as the PA derivative. A triacylglycerol (1,2-Dilinoleoyl-3-palmitoyl-*rac*-glycerol) with a palmitoyl tail at the sn-3 position also increases significant fluorescence overlap (Figure 6). . These results indicate that the PA derivatives possibly promote membrane aggregation and/or fusion.

**Figure 7:**
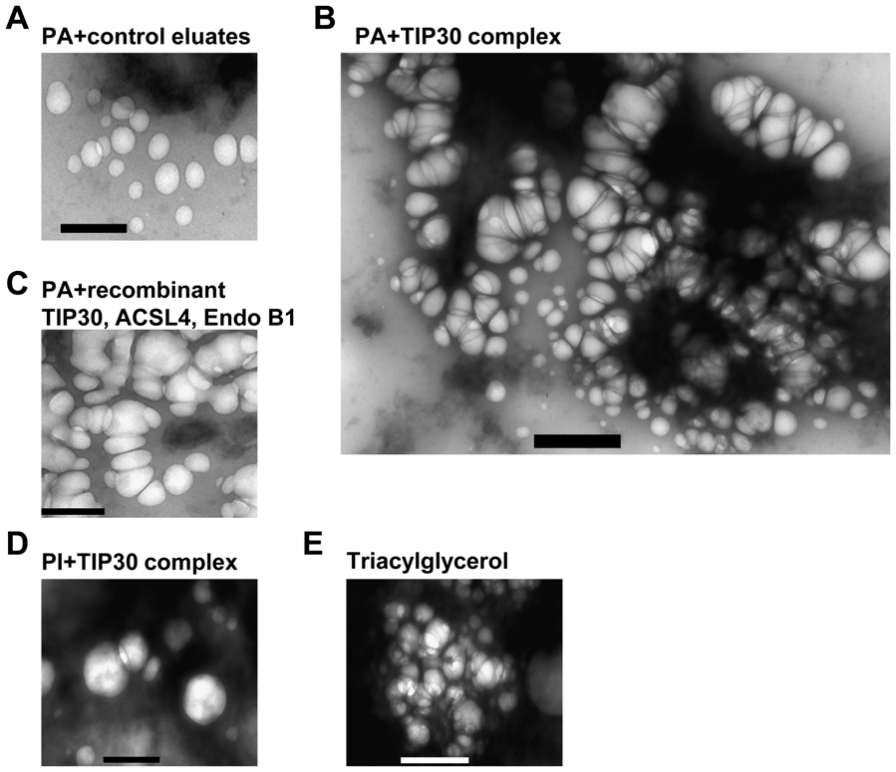
Fatty acylation of phosphatidic acid induce vesicle tethering and stacking. Effects of acylated PA on vesicle fusion were determined using electron microscope. Lipids were extracted after incubating PA with control immunoprecipitates (A), PA with immunopurified TIP30 complex (B), PA with recombinant TIP30, ACSL4, and Endo B1 (C) or PI with immunopurified TIP30 complex (D). Each of these lipids or triacylglycerol (E) was suspended in homogenization buffer and incubated on ice with EGFR-DsRed and EYFP-Rab5a vesicles. The resulting vesicles were examined using TEM. Scale bars, 500 nm.

Finally, we used electron microscopy to examine the vesicles after incubation with PA derivatives that were generated by either immunopurified or recombinant protein-reconstituted TIP30 complexes (Figure 7). PA derivatives caused vesicle tethering and stacking, which is marked by dramatic deformation (Figure 7B and 7C). In contrast, the vesicle aggregation caused by triacyglycerol seemed due only to tethering (Figure 7E), indicating that these specific commercially available triacyglycerols do not have the ability to induce vesicle stacking; therefore do not represent the new lipid species generated by the TIP30 complex. Consistent with the confocal microscopy data, PA treated with control eluates (Figure 7A) or PI treated with immunpurified TIP30 complex (Figure 7D) had no apparent effect.

Nonetheless, we did not observe complete fluorescence overlap and enlarged endosomes (>0.5 µm in diameter) as seen in Figure 2, suggesting that additional activities of the TIP30 complex or other proteins on the membranes, such as SNAREs, SNARE accessory factors, Rab5a and its effectors, are needed to accomplish the fusion steps following close membrane contact. Collectively, these data indicate that TIP30, ACSL4, and Endo B1 promote vesicle membrane fusion by fatty acylating PA.

## Discussion

Elucidating the molecular basis of intracellular trafficking and protein sorting requires the identification of the critical components participating in these processes. Our earlier studies demonstrated that proteins in the TIP30 protein complex are required for the fusion of endocytic and Rab5a vesicles *in vivo*. In the present study, using both confocal microscopy and transmission electron microscopy, we demonstrated that the TIP30 complex promotes membrane fusion *in vitro* and that arachidonyl-CoA synthesis by ACSL4, a component in the TIP30 complex, is essential for membrane fusion.

Arachidonic acid has been shown to be involved in vesicle membrane fusion [6], [13]. Previous reconstituted approaches *in vitro* have contributed greatly to our understanding of membrane fusion. However, to our knowledge, efficient fusion between biological membranes has hardly been achieved *in vitro* in cell free assays without using cytosol fractions or arachidonic acid, further emphasizing the important role of arachidonic acid during membrane fusion. How does arachidonic acid promote membrane fusion? Arachidonic acid has been proposed to stimulate SNARE complex assembly [21], [22], [23]. Our data seem support these observations by showing that endocytic vesicles recognize Rab5a vesicles only after arachidonic acid was added to the reactions. Interestingly, fusion did not occur in the absence of the TIP30 complex or in the presence of the ACSL4 inhibitor. These results suggest that in addition to directly promoting SNARE complex assembly, arachidonic acid must be activated by esterification to stimulate membrane fusion.

We further showed that the arachidonyl group is transferred to endosomal PA to generate a new lipid species that induce vesicle tethering and stacking. Thus, we suggest a hypothesis that arachidonic acid promotes membrane fusion by contributing to both PA acylation and SNARE complex assembly. Consistent with this view, PA and its synthetase phospholipase D (PLD) are known to participate in membrane fusion during vesicle transport [20], [24]. Moreover, as integral components of many biological membranes, triacylglycerols have been demonstrated to possess great potency to promote spontaneous curvature in an acyl chain length dependent manner [25], which may alter membrane structure to support membrane fusion [1], [2]. We speculate that the addition of the hydrophobic acyl chain to endosomal PA may help to overcome the repulsive hydration force generated from water bound to the lipid headgroups. In addition, triacylglycerols can exhibit an extended conformation [26] with the 3-arachidonyl group in the opposite direction of the other two acyl chains, thereby allowing for attachment and fusion between two membranes [27], [28]. Our results suggest that as an initiation event, acylation of endosomal PA enables the close contact between donor and recipient membranes, thus allowing for fusion to be accomplished by SNAREs, SNARE accessory factors, Rab5 and its effectors. Nevertheless, although we favor this hypothesis to explain the role of TIP30 in membrane fusion and endocytic trafficking, we do not rule out other possible hypotheses that may also explain the actions of the TIP30 complex. Undoubtedly, efficient fusion could be achieved using artificial membranes in the absence of arachidonic acid. This may be due to the fact that artificial membranes are far less complex than biological membranes in terms of membrane composition, organization and dynamics, thereby requiring much less factors to fuse. This view is supported by the observation that the same set of proteins could promote efficient fusion of artificial membranes but incapable of inducing efficient biological membrane fusion [12]. Furthermore, our data suggest that the TIP30 complex modifies lipids to initiate membrane fusion. This step may have been bypassed during the processes of lipid extraction and membrane reconstitution. It would be interesting to find out whether addition of arachidonic acid, CoA and the TIP30 complex could enhance artificial membrane fusion.

Clearly, more work is needed to determine how fatty acylated PA and other lipid derivatives cooperate with the action of SNAREs, Rab5 and their effectors in membrane fusion. Future studies may also focus on finding out how the TIP30 complex acylates PA and what the exact identity of the fusion products is. It is expected that a more precise mechanism underlying membrane fusion will emerge by integrating information from studies of both lipids and proteins.

## Materials and Methods

### Reagents

DMEM, fetal bovine serum, trypsin, penicillin, and streptomycin were purchased from Invitrogen. Anti-HA agarose beads were from Roche Applied Science. Polyclonal rabbit anti-human Rab5a antibody was from Cell Signaling. Polyclonal rabbit anti-human ACSL4 was a generous gift from Stephen Prescott (University of Utah). Polyclonal rabbit anti-human Endo B1, LPA, triacylglycerol (1,2-Dilinoleoyl-3-palmitoyl-*rac*-glycerol), arachidonic acid and CoA were from Sigma-Aldrich. PA (1,2-dioleoyl-sn-glycero-3-phosphate) were purchased from Avanti Polar Lipids. [^3^H]-arachidonic acid was from PerkinElmer.

### Cell culture

PLC/PRF/5 and HepG2 cell lines were purchased from ATCC and cultured in DMEM supplemented with 10% fetal bovine serum and penicillin/streptomycin at 37°C in 5% CO_2_.

### Immunoprecipitation

Immunoprecipitations were performed as previously described [14].

### Purification of endocytic and Rab5a vesicles

HepG2 cells were transduced by the lentivirus carrying vector pSin-EGFR-DsRed or pSin-EYFP-Rab5a and were selected for 4 days with 2 µg/ml puromycin. To prepare endosomes carrying EGFR-DsRed, we treated cells with 10 ng/ml EGF at 37°C for 10 minutes and purified early endosomes on the flotation gradient essentially as described [29]. Briefly, cells were incubated with 10 ng/ml EGF for 10 min at 37°C. After several washings, cells were scraped and pelleted at 4°C before being subjected to needle breakdown in homogenization buffer (250 mM sucrose, 3 mM imidazole, pH 7.4, 1 mM EDTA) at 4°C. Homogenates were centrifuged at 3000 rpm for 10 min at 4°C and post-nuclear supernatants (PNS) were collected. PNSs were then suspended in 40.6% sucrose by adding a stock solution (62% sucrose, 3 mM imidazole, pH 7.4, 1 mM EDTA) and loaded at the bottom of a SW40 centrifugation tube. The load was then sequentially overlaid with 1.5 ml of 35% sucrose in 3 mM imidazole, pH 7.4 with 1 mM EDTA; 1 ml of 25% sucrose in 3 mM imidazole, pH 7.4 with 1 mM EDTA; and at the top with 0.5 ml of homogenization buffer. The gradient was centrifuged at 125000 g for 60 min at 4°C. Early endosomal fractions were collected at the 35%/25% sucrose interface.

Rab5 vesicles containing EYFP-Rab5a were prepared as described [30]. Briefly, HepG2 cells expressing pSin-EYFP-Rab5a were starved for 24 hours before being scraped and pelleted at 4°C. PNSs were prepared as described above. PNSs were then centrifuged at 10000 rpm at 4°C using a bench top centrifuge. The post mitochondria supernatants were loaded on top of homogenization buffer in SW40 centrifugation tubes with 0.5 ml cushion solution (62% sucrose, 3 mM imidazole, pH 7.4, 1 mM EDTA) at the bottom. Centrifugation was done at 100000 g for 60 min at 4°C and Rab5a vesicles were collected on top of the cushion solution.

### *In vitro* vesicle fusion assay

The assay was performed as described previously with modifications [16], [17]. Briefly, EGFR-DsRED endocytic vesicles and EYFP-Rab5a vesicles (aliquotes of both contain 20 µg proteins) were gently mixed in a total volume of 20 µl fusion buffer (10 mM Hepes, pH 7.4, 1.5 mM MgOAc, 1 mM DTT, 50 mM KOAc, 100 nmol arachidonic acid, 1 mM coenzyme A, 5 mM GTP, complemented with 4 µl of an ATP-regenerating system containing 1∶1∶1 mixture of 100 mM ATP, 600 mM creatine phosphate, and 4 mg/ml creatine phosphokinase). After incubating with indicated purified proteins at 37°C for 40 min, a portion of the reactions were spotted on slides and examined using Zeiss LSM 510 Meta confocal microscope. All images are representative single optical sections. Colocalization analysis was done using MBF_ImageJ. For reactions in Figure 5, PA and PI were incubated with control eluates or the TIP30 complex in a total volume of 200 µl fusion buffer. Resulting lipids were purified using Bligh-Dyer Method [31] and incubated with vesicles in homogenization buffer (250 mM sucrose, 5 mM Hepes, pH 7.4) on ice. Electron microscopy studies was performed as described previously using TEM JEOL 100CX [12].

### Protein-lipid overlay assays

Protein-lipid overlay assays were performed essentially as previously described [32]. Briefly, membrane strips (Echelon Biosciences Inc.) containing 15 pre-spotted lipids were incubated overnight at 4°C with recombinant proteins (10 µg/ml) in TBST with 5% milk. After being washed with TBST 10 min each for 3 times, the strips were incubated overnight at 4°C with specific primary antibodies against the recombinant proteins in TBST with 5% milk. The strips then were washed again and incubated with corresponding fluorescent secondary antibodies at room temperature for 1 hour. Images were acquired by scanning the strips using a Li-Cor scanner.

### Lipid acylation

Purified endocytic vesicles (aliquots containing 20 µg proteins) were incubated with indicated proteins and [^3^H]-arachidonic acid (PerkinElmer) in the presence or absence of 10 µM triacsin C at 37°C for 1 hour in a total volume of 200 µl fusion buffer. Lipids were extracted using Bligh-Dyer Method [31] and resolved on silica gel 60 TLC plate with chloroform/ethanol/water/triethylamine (30/35/7/35) as the solvent. The TLC plate was exposed to Kodak Tritium Sensitive Storage Phosphor Screen. Images were acquired by scanning the screen using a Molecular Dynamics Storm 860. For preparation of lipid derivatives from PA, PI or LPA, 100 nmol lipids were incubated with immunopurified TIP30 complex or recombinant TIP30, ACSL4 and Endo B1 in the fusion buffer. Resulting lipids were purified using Bligh-Dyer Method [31].

### MS/MS spectrometry analysis

MS/MS spectrometry analysis of PA derivatives were performed in Avanti Polar Lipids Inc. Briefly, PA derivatives were extracted according to Bligh-Dyer method and redissoved in methanol/chloroform (85/18) with 10 mM NH_4_OAC and 1 ug/ml NH_4_OH. First, standards of 17:0–20:4 PA and 17:0–20:4 PI were prepared in the above solution and infused in the API 4000 QTrap triple quadrupole with linear ion trap instrument for optimization of collisional energy to provide mass fragments of arachidonic acid (20:4) at 303.2 u. Then samples were flow injected into the MS/MS in negative scan mode to discover products from the described reaction. The m/z value was used to search the most likely molecular species at http://www.lipidmaps.org.

### Statistical analysis

All statistical tests were two-tailed t-test. Data represent means ± SEM. *P<0.05, **P<0.01.
